# “Pulmonary Actinomycosis attributable to *Actinomyces meyeri* presenting as cardiac tamponade: a case report”

**DOI:** 10.1186/s40248-018-0132-9

**Published:** 2018-06-14

**Authors:** Saori Nishizawa, Keisuke Anan, Kazunori Tobino, Masanobu Okahisa, Yuki Goto, Kojin Murakami, Takuto Sueyasu, Miyuki Munechika, Kohei Yoshimine, Mai Yoshino

**Affiliations:** 1 Department of Respiratory Medicine, Iizuka Hospital, 3-83 Yoshiomachi, Iizuka, Fukuoka, 820-0018 Japan; 2 Department of Respiratory Medicine, Saiseikai Kumamoto Hospital, 5-3-1 Chikami, Minami-Ku, Kumamoto, 861-4193 Japan

**Keywords:** *Actinomyces meyeri*, Cardiac tamponade, Lung abscess, Actinomycosis

## Abstract

**Background:**

Recently, it is reported that *Actinomyces meyeri* is the most common species causing actinomycosis. However, to our knowledge, there was no report about pulmonary actinomycosis attributable to *A. meyeri* presenting as cardiac tamponade.

**Case presentation:**

Hereby we describe a case of pulmonary actinomycosis attributable to *A. meyeri* presenting as cardiac tamponade. At first, the patient was diagnosed with bacterial pericarditis with lung abscess in the left lower lung lobe and underwent pericardial drainage. Three days after the removal of the drainage tube, atrial fibrillation followed by cardiac arrest with asystole occurred and immediate cardiopulmonary resuscitation restored his circulation. Thereafter, he underwent pneumocentesis of the lung abscess and the culture grew *A. meyeri*. He was successfully treated with penicillin G.

**Conclusion:**

This is the first case of pulmonary actinomycosis attributable to *A. meyeri* presenting as cardiac tamponade. We believe that an increased awareness of the disease is necessary to expedite diagnosis therefore minimizing morbidity and mortality.

## Background

Until recently, it was thought that *Actinomyces israelii* was the most common cause of actinomycosis, and *A. meyeri* was a rare causative agent. However, recently, Rolfe R et al. reported that *A. meyeri* might be a more common cause of actinomycosis than previously recognized [1]. In their report, *A. meyeri* was more common than *A. israelii* in 130 patients (43 and 30%, respectively) who had a positive result for *Actinomyces* cultures with adequate follow up in the Medical University of South Carolina between 1988 and 2016.

Actinomycosis is an indolent, slowly progressive bacterial infectious disease due to *Actinomyces* species [2]. Actinomycosis usually involves the cervicofacial (55%), abdomino-pelvic (20%), thoracic (15%), and mixed organ involvement (10%) including the skin, brain, pericardium and, extremities [3]. *Actinomyces* species are one of the normal bacterial flora of man’s oral cavity, and aspiration, trauma and poor dental hygiene are predisposing factors for initial infection [4–7]. It was reported that infections due to *A. meyeri* were more prevalent among males (the male-to-female ratio was 20:6), the mean age of patients was 42.5 years (range, 13–70 years), and dentogingival disease was a major risk factor for the development of infections, as with other actinomycoses [8, 9].

*A. meyeri* are prone to disseminated actinomycosis (defined as the involvement of two distant organs) [8]. But, to our knowledge, there was no report about pulmonary actinomycosis attributable to *A. meyeri* presenting as cardiac tamponade.

## Case presentation

**Fig. 1:**
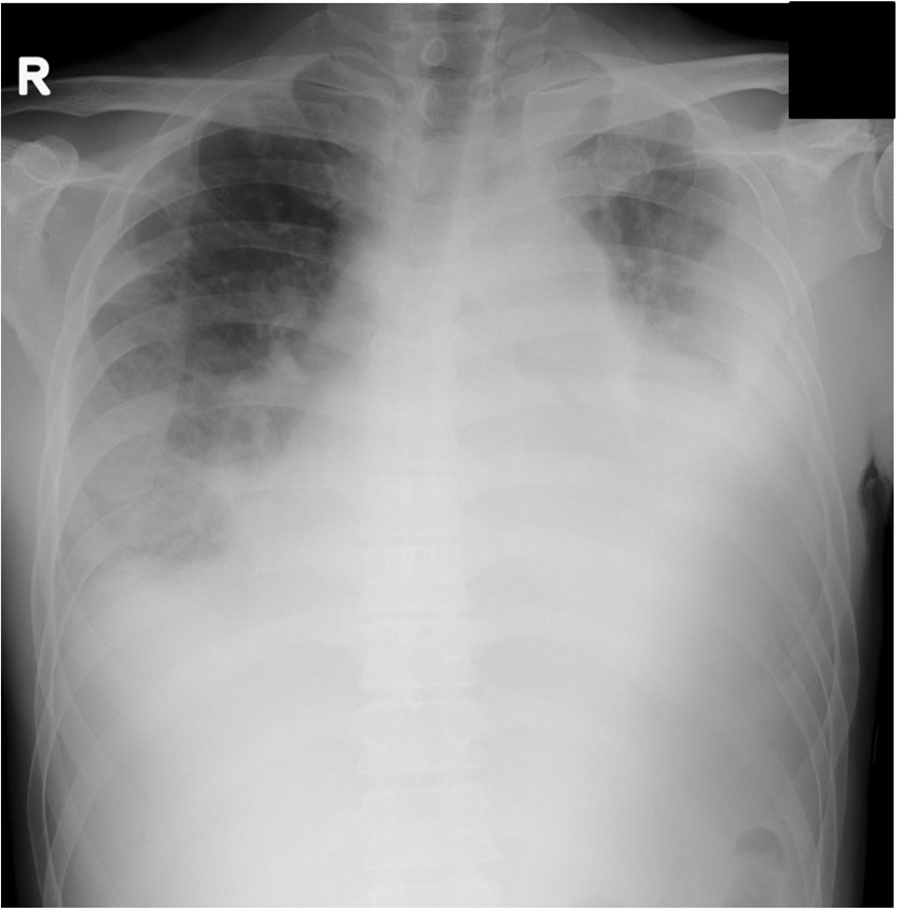
The chest radiograph revealed a mass in the left upper lung, bilateral pleural effusions and cardiac enlargement

**Fig. 2:**
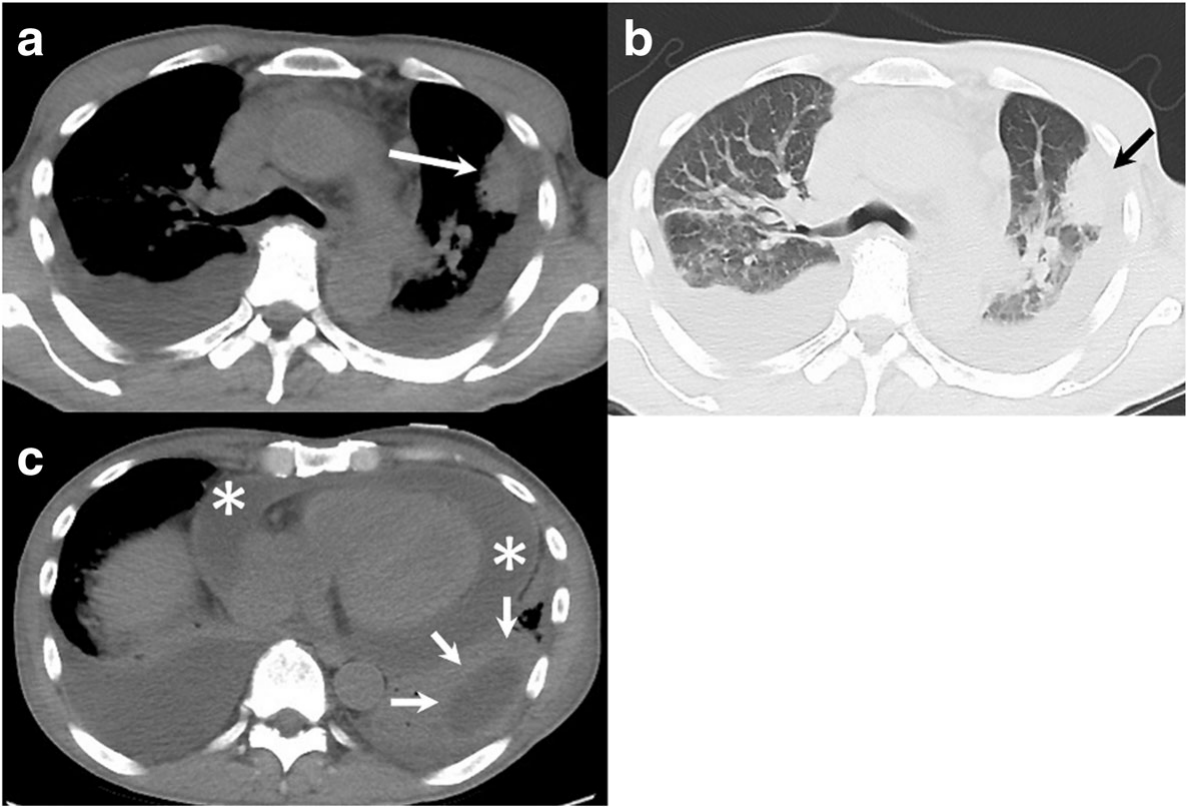
The chest CT scan showed a wedge-shaped and pleural-based mass in the left upper lobe (white arrow on (**a**), and black arrow on (**b**)), thick-walled cavitary lesion containing water density in the left lower lobe (white arrows on (**c**)), bilateral pleural effusions and pericardial effusion (* on (**c**))

A 56-year-old Japanese male was referred to our hospital with dyspnea and hypotension. He had a history of Parkinson’s disease with psychosis. He complained of left-sided chest pain and productive cough in the 2 months before admission. He reported no risk factors for HIV infection, occasional alcohol consumption, and was a current smoker with a 30-pack/year smoking history. Initial vital signs were as follows: blood pressure, 77/56 mmHg; heart rate, 106/min; body temperature, 36.1 °C (97.0 °F). There was no lymphadenopathy or hepatosplenomegaly. Examination of the oral cavity revealed poor dentition and inadequate hygiene. Jugular venous pressure was elevated to the angle of the jaw at 45° and did not change with respirations. The breath sounds attenuated at both sides, and dullness was present at the base of the bilateral lung. Cardiovascular examination revealed regular rhythm, tachycardia, and distant heart sounds. Laboratory test values were as follows: white blood cells, 29,950/mm^3^ with a left shift; hemoglobin, 10.6 g/dl; platelets, 453,000/mm^3^; random serum glucose, 125 mg/dl (normal, 75–115 mg/dl); serum lactate dehydrogenase (LDH), 260 U/l (normal,119–229 U/l); serum aspartate and alanine aminotransferase (AST and ALT), 80 U/l and 24 U/l (normal, 0–35 U/l); serum albumin, 3.1 g/dl (normal, 4–5 g/dl); serum C-reactive protein (CRP), 17.25 mg/dl (normal, < 0.2 mg/dl). The chest radiograph (Fig. 1) revealed a mass in the left upper lung, bilateral pleural effusions and cardiac enlargement. The chest computed tomography (CT) scan showed a wedge-shaped and pleural-based mass in the left upper lobe (LUL), a thick-walled cavitary lesion containing only water density in the left lower lobe (LLL), bilateral pleural effusions and pericardial effusion (Fig. 2). An electrocardiogram was normal. The patient received acute pericardiocentesis and pericardial drainage tube placement, which yielded approximately 800 ml of dark yellow fluid and restored blood pressure. Thoracentesis on both sides was performed and revealed yellow and turbid pleural fluid. Gram stain and cytologic examination of both pericardial and pleural fluid showed no organisms and also malignant cells. Examination of sputum showed no pathogen on staining. Two sets of blood culture specimens drawn at the time of admission did not yield any organisms. The patient was initially diagnosed with a bacterial pericarditis and lung abscess in the LLL. Intravenous ceftriaxone was started as an empirical antimicrobial treatment.

**Fig. 3:**
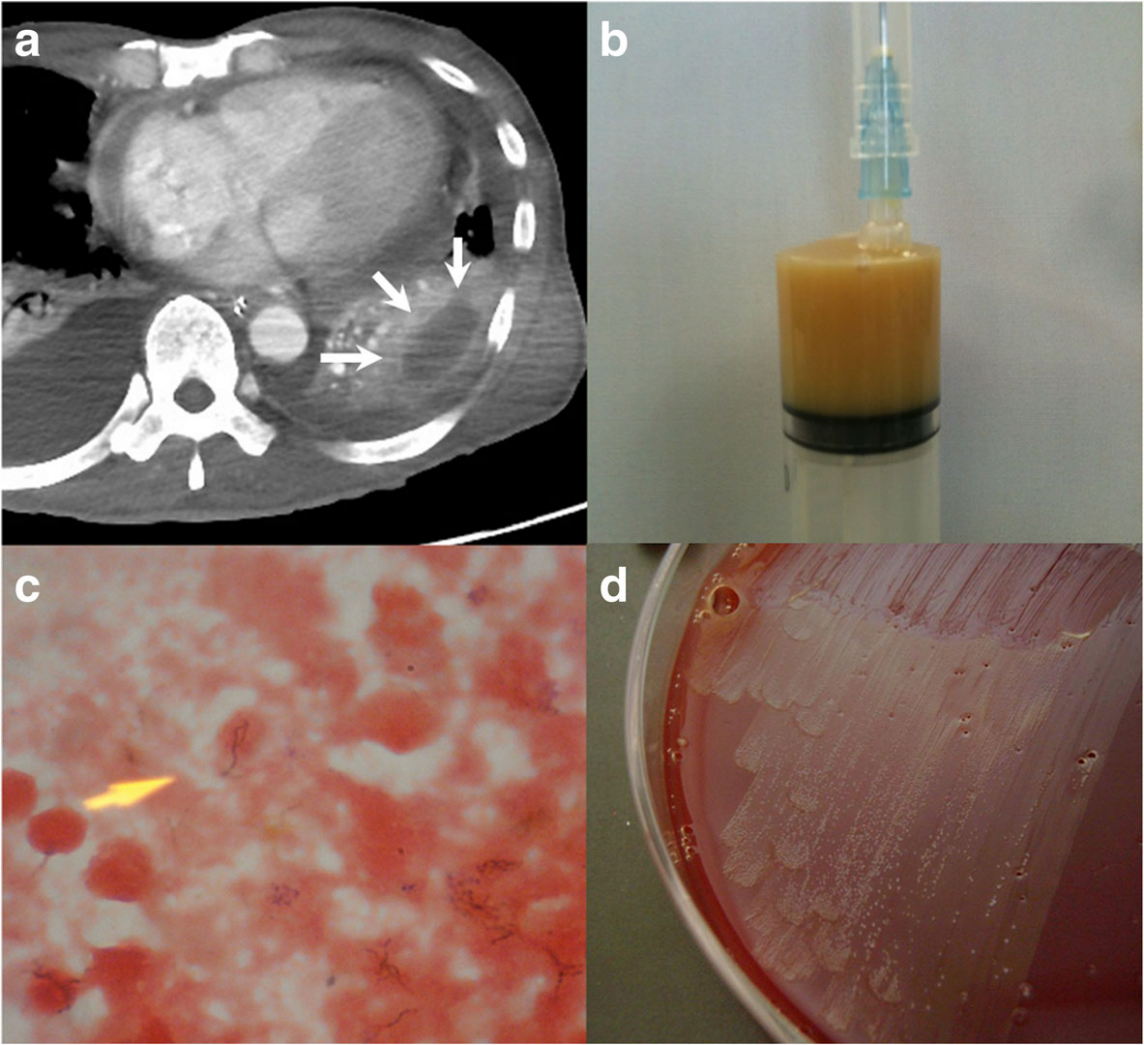
**a** The chest CT with enhancement on the 9^th^ day showed a thick-walled cavitary lesion containing water density in the left lower lobe (white arrows) and a small amount of pericardial effusion; **b** Purulent fluid obtained by ultrasound-guided pneumocentesis of the cavitary lesion in the left lower lobe; **c** Gram stain of the fluid showed Gram-positive filamentous rods; **d** Cultures from the fluid grew *Actinomyces* species

**Fig. 4:**
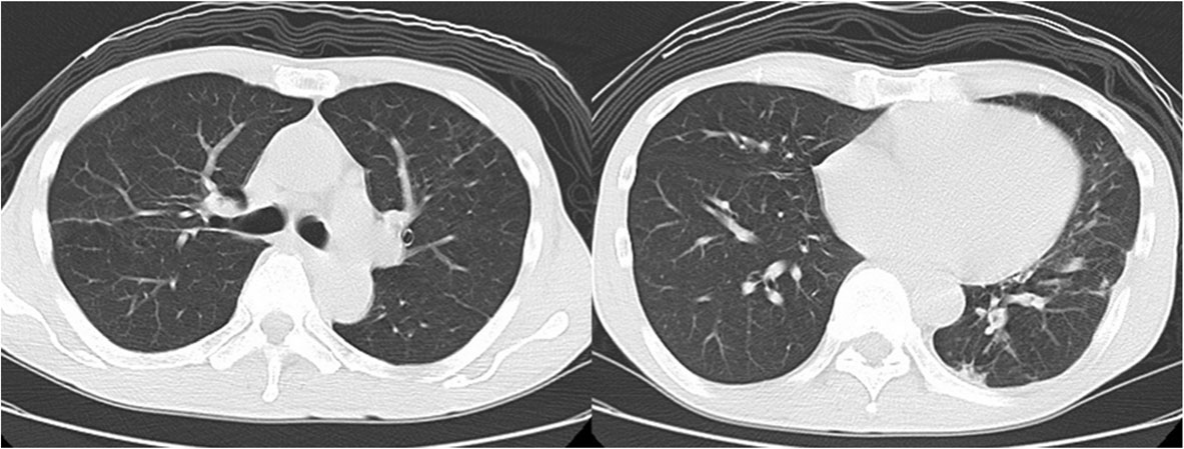
The chest CT scan after the completion of six-month antibiotic therapy showed a significant improvement

After the admission, the patient continued to be afebrile and his respiratory and cardiovascular status was stable. On day 6, there was minimal pericardial fluid in the drainage tube so the drain was removed. In the morning of day 9, a fever of 39.0 °C (102.2 °F) and sinus tachycardia (150/min) occurred. The tachycardia continued for 4 h, and he experienced atrial fibrillation (Af). Immediately after the onset of Af, a cardiac arrest with asystole occurred. He underwent an immediate cardiopulmonary resuscitation, which restored sinus rhythm and blood pressure. Chest CT scan obtained on the same day revealed the remaining thick-walled cavitary lesion in the LLL and the reduced pericardial effusion. After the cardiopulmonary state was stabilized, he underwent ultrasound-guided pneumocentesis of the cavitary lesion in the LLL and 35 ml of purulent fluid was obtained (Fig. 3). Gram stain of the fluid revealed Gram-positive filamentous rods, and cultures of the fluid grew *Actinomyces* species (Fig. 3). We analyzed the fluid using a method for clone library sequencing of the 16S ribosomal DNA (rDNA) gene and *Actinomyces meyeri* along with other anaerobes (*Fusobacterium* species) were detected [10]. Transbronchial biopsy and bronchial washings of the mass lesion in both the LUL and LLL were performed. The biopsy revealed non-specific inflammation and organization of the lung tissue with no bacteria. On day 11, antibiotics were changed to intravenous penicillin, and his condition continued to be stable. After 4 weeks of intravenous penicillin therapy, antibiotics were switched to oral doxycycline therapy and he was discharged. Echocardiogram before the discharge showed no evidence of pericardial effusion or constrictive physiology. The patient completed the total six-month antibiotic therapy. At follow up, 6 months after discharge, the patient was gaining weight, felt well, and his CT images had continued to show improvement (Fig. 4).

## Discussion

To our best knowledge, this is the first case report of pulmonary actinomycosis attributable to *A. meyeri* presenting as cardiac tamponade.In our patient, the pericarditis seemed to be caused by the direct extension of the inflammation from pulmonary actinomycosis, because we could not detect any microorganisms in the patient’s pericardial fluid. The arrhythmia which suddenly occurred in our patient and did not recur after the penicillin therapy suggests cardiac actinomycosis, however, evidence was not obtained.

Cardiac actinomycosis is very rare, and the frequency of this condition was reported as only 1.2 to 2% of all cases of actinomycosis [11, 12]. Cardiac actinomycosis was first reported in 1884 [13], and to date, two comprehensive reviews about this condition have been published. Cornell et al. documented 68 cases of cardiac actinomycosis and Fife et al. detailed 19 cases of pericardial actinomycosis documented [11, 14]. Cardiac involvement was usually caused by the direct extension of thoracic disease [15, 16]. In fact, it was reported that 23 out of 29 cases (79%) of pericardial actinomycosis since 1950 had thoracic involvement [17]. It is characterized by involvement of the pericardium, myocardium, and endocardium in a decreasing order of frequency, which is consistent with its mode of contiguous spread through tissue planes from the thorax [18]. Our patient had pulmonary lesions as primary sites of infection, and the pericardial involvement seemed to be caused by the direct extension from the primary sites However, in our patient, pericardial fluid did not show the evidence of *A. meyeri* infection. Actually, the diagnosis of actinomycosis is generally hampered by the difficulty in isolation and culture of the organism. It must be cultured in strictly anaerobic conditions. In the review of Fife et al., purulent pericardial fluid was obtained from 10 (53%) of 19 cases, however, *Actinomyces* species were successfully cultured from the fluid in only two of those 10 cases [14]. Similarly, low yields (26%) of biopsy specimen cultures have been observed [14]. Sulfur granules, long regarded as a histological hallmark of actinomycosis, are very strongly suggestive of the diagnosis. However, they are not entirely specific to actinomycosis, since these granules can also be found in nocardiosis, botryomycosis, aspergillosis, and coccidioidomycosis [4, 19]. Fortunately, gram stain of the purulent fluid obtained by pneumocentesis showed Gram-positive filamentous rods, and cultures from the purulent fluid grew *Actinomyces* species. Finally, the genetic testing with a method for clone library sequencing of the 16S rDNA gene of the fluid revealed *A. meyeri* along with *Fusobacterium* species. It was reported that two-thirds of the infections with *A. meyeri* were polymicrobial [8], and it was thought that reduced oxygen tension and inhibition of phagocytes induced by concomitant bacterial infections might enhance the pathogenicity of *Actinomyces* species [9].

In patients with cardiac actinomycosis, the prognosis is good when correct diagnosis and medical treatment are made, and appropriate choice of antibiotics and pericardial drainage are important. It was reported that when the diagnosis of cardiac actinomycosis was given and appropriate antibiotic therapy was started, 86% of patients survived [17]. The recommended antibiotic therapy is high doses of intravenous penicillin G (12–20 MU/d in divided doses) for 4 to 6 weeks, followed by 6 to 12 months of oral penicillin/amoxicillin [20]. Alternative choices in patients allergic to penicillin are sulfonamide, tetracycline, erythromycin, or third-generation cephalosporin. *A. meyeri* is also susceptible to most antibiotics, and penicillin remains the most cost-effective drug. The associated bacteria are generally susceptible to the antibiotics for the treatment of *A. meyeri.* According to the previous reports, a pericardial drainage or pericardiectomy was required in 82 and 45% of surviving patients, respectively. Our patient was successfully treated by pericardial drainage, pneumocentesis, and antibiotics.

## Conclusion

To our knowledge, this is the first case who survived pulmonary actinomycosis attributable to *A. meyeri* presenting as cardiac tamponade. We believe that an increased awareness of the disease is necessary to expedite diagnosis therefore minimizing morbidity and mortality.

## Abbreviations

Af: Atrial fibrillation
AST and ALT: Aspartate and alanine aminotransferase
CRP: C-reactive protein
CT: Computed tomography
LDH: Lactate dehydrogenase
LLL: Left lower lobe
LUL: Left upper lobe
rDNA: Ribosomal DNA
